# Preparation of MXene/BN Composites with Adjustable Microwave Absorption Performance

**DOI:** 10.3390/ma16206752

**Published:** 2023-10-18

**Authors:** Weidong Zhang, Haoliang Wen, Yaping Gou, Yun Zhao, Zhiqiang Zhang, Yali Qiao

**Affiliations:** 1 College of Chemical Engineering, Qinghai University, Xining 810016, China2017990112@qhu.edu.cn (Y.Z.);; 2 State Key Laboratory of Plateau Ecology and Agriculture, Qinghai University, Xining 810016, China

**Keywords:** microwave absorbents, high temperature resistant, BN nanosheets

## Abstract

The challenge of developing a high-efficiency microwave absorbent remains, because of the compatibility between microwave absorption and high-temperature-resistant performance in practical application. Herein, a facile method is used to obtain serial MXene/BN-zxy composites, where zx:y indicates the weight ratio of MXene and boron nitride (BN) in the composites, with adjustable microwave absorption performance (MAP) which can be regulated by the ratio of MXene and the BN nanosheet. In particular, the as-prepared absorbents with supercapacitance-like structure significantly enhanced the MAP and could be served more than 900 °C. The results of MAP reveal that the minimum reflection loss (RL) can reach −20.94 dB with a MXene/BN-101 composite coating thickness of 4.0 mm; the effective attenuation bandwidth (RL< −10 dB, i.e., 90% microwave energy is attenuated) is up to 9.71 GHz (7.94–17.65 GHz). From a detailed analysis, it is observed that attenuation is the critical limiting factor for MAPs rather than impedance mismatch, which can be assigned to the poor MAP of BN nanosheets. In any case, as-prepared absorbents have potential applications in the field of heating components.

## 1. Introduction

With the advancement of the electronic industry, various equipment has significantly improved the quality of people’s lives [1,2,3], simultaneously leading to potential risks for human health and safety in the commercial and military sectors as a result of the proliferation of electromagnetic (EM) waves [4,5,6]. Thus, a drastic measure was adopted by the research to prevent EM wave radiation. Recently, two-dimensional (2D) materials such as, graphene [7,8,9], MoS_2_ [10,11,12] and MXene [13,14,15] have come to be regarded as the first candidates for microwave absorbents, based on their inherent physical and chemical properties, with light weight, high porosity and muti-interface characterization. However, absorbents with low density, highly effective absorption and high temperature resistant performance are facing research in practical applications.

To construct the abovementioned ideal absorbents, the researcher should pay attention to inorganic ceramic materials based on the MXene, such as ZnO [16,17,18], SiC [19,20,21], MnO_2_ [22,23,24], CeO_2_ [25,26,27] and TiO_2_ [28,29,30]. The preparation of an urchin-like ZnO/Ti_3_C_2_Tx nanocomposite was described by Qian et al. [31] through a coprecipitation process. The conclusion demonstrated that the optimized RL value was −26.30 dB, which significantly outperformed the original Ti_3_C_2_T_x_. The Ti_3_C_2_T_X_-MXene compound beaded SiC nanowire membrane was prepared using a simple extraction and filtration technology by Wang et al. [32]. With a thickness of 1.58 mm, the minimum RL value reached −41.7 dB, corresponding to an actual absorption width of 3.36 GHz. The Ni-MnO_2_/MXene composites were fabricated by incorporating Ni-doped MnO_2_, presenting a promising choice for oxygen electrode materials in the study conducted by Wu et al. [33]. As expected, these composites exhibited an RL value of −55.9 dB and an absorption bandwidth of 6.32 GHz. Liu et al. [34] successfully employed a thermal method to fabricate exquisite 2D MXene-based composites incorporating CeO_2_ nanoparticles. Remarkably, the nanocomposite of CeO_2_/MXene showcased an astonishingly low RL value of −47.27 dB at a mere thickness of 1.9 mm, thereby demonstrating its exceptional efficacy in absorbing electromagnetic waves across an extensive frequency range, reaching up to 4.22 GHz. The MXene@TiO_2_ composite was prepared using a simple hydrothermal oxidation method [35]. It exhibited an RL value of −0.3 dB at a thickness of 1.75 mm, and the maximum effective attenuation width was 4.08 GHz with a thickness of 1.26 mm.

Normally, the microwave absorbents act as a coating to prevent the EM wave from radiating. Thus, the thermal conductivity must be taken into account, especially when used in the field of heating components. Concerning the compatibility between high temperature resistance and thermal conductivity, boron nitride (BN) impresses me strikingly as having excellent thermal conductivity compared with all the inorganic materials and by being able to be synthesized at 900 °C by borate acid and urea. Shi et al. [36] utilized the in situ synthetic process to fabricate a TiN composite exhibiting a minimum RL of −16.74 dB at 873 K and demonstrated robust high thermal attenuation characteristics in the X-band with 3.26 GHz. Jiang et al. [37] employed a one-stage thermal nitridation method to produce BN/RGO nanocomposites. The results indicated that the composites achieved an impressive RL value of −48.9 dB over a broad effective absorption bandwidth of 4.2 GHz, with a low filler content of 30 wt.% and at a thickness of 2.6 mm.

Therefore, in this paper, serials of MXene/BN nanocomposites were synthesized using a facile reaction in the presence of borate acid and urea. The microwave absorption performance of the as-prepared sample was significantly related to the ratio of MXene and BN nanosheets. More importantly, MXene/BN nanocomposites with supercapacitance-like structure were constructed with the in situ calcination method to realize the compatibility between microwave absorption and high-temperature-resistant performance. In addition, the micro-structure (SEM and TEM), elements composition, impedance matching and attenuation performance were systematically explored and cleared out. Finally, all the related MXene or BN absorbents are summarized in Table 1.

## 2. Materials and Methods

### 2.1. Materials

The reagents were utilized as received without any additional purification. Boracic acid (H_3_BO_3_, A.R.) was procured from Shanghai Aladdin Biochemical Technology Co., Ltd. Urea (CO(NH_2_)_2_, A.R.) (Shanghai, China) and was supplied by Guangdong Guanghua Sci-Tech Co., Ltd. (Shantou, China). MAX power (Ti_3_AlC_2_) with an average diameter of 400 meshes was provided by Forsman Scientific (Beijing) Co., Ltd. (Beijing, China). Hydrofluoric acid (HF, 40%) was provided by Shanghai Macklin Biochemical Co., Ltd. (Shanghai, China).

### 2.2. The Preparation of MXene (Ti_3_C_2_T_x_)

The lamellar MXene materials were prepared by etching MAX powder with 40% HF. Firstly, 1 g Ti_3_AlC_2_ was added in 20 mL HF solution with a temperature of 0 °C. After magnetic stirring for 48 h at 0 °C, the product was collected via centrifugation, and was washed with deionized water several times until the appropriate value of pH (pH ≥ 6) was reached. The prepared MXene powder was obtained by freeze drying for 48 h. The specific equation can be obtained from the literature [44].

### 2.3. The Preparation of MXene/BN Composites

The MXene/BN hybrids were fabricated via chemical vapor (CVD). Appropriate amounts of MXene ensured that weight proportions of precursor MXene/BN at ratios of 10:1, 20:1 and 25:1. H_3_BO_3_ (0.062 g) and CO(NH_2_)_2_ (2.98 g) were dissolved in ultrapure water. As-prepared MXene (0.25 g) was dispersed in the solution mixture through sonication for 30 min. After magnetic stirring for 24 h, the precursor was freeze-dried and then annealed at a temperature of 900 °C for 5 h under Ar atmosphere protection in a tube furnace.

The decomposition of CO(NH_2_)_2_ and the production of NH_3_ were observed at 160 °C, as shown in Equations (1)–(3). At 180 °C, H_3_BO_3_ started to decompose and generate B_2_O_3_, which then began to melt at 450 °C. Due to the extensive gas–liquid contact area, the molten B_2_O_3_ attached to the MXene sheets absorbed a significant amount of NH_3_ for participation in the reaction. It was not until the temperature reached 900 °C that boron nitride crystals began to grow perfectly within the layers of MXene. Upon cooling down to room temperature, MXene/BN hybrids were obtained. The composites with different ratios (25:1, 20:1, 10:1) of MXene and BN were named MXene/BN-251, MXene/BN-201 and MXene/BN-101, respectively.

(1) $$6{\mathrm{NH}}_{2}{\mathrm{CONH}}_{2}\overset{\mathrm{Heat}}{\to }6{\mathrm{NH}}_{3}+C_{3}H_{6}N_{6}+3{\mathrm{CO}}_{2}$$

(2) $$2H_{3}{\mathrm{BO}}_{3}\overset{\mathrm{Heat}}{\to }B_{2}O_{3}+3H_{2}O$$

(3) $$B_{2}O_{3}+2{\mathrm{NH}}_{3}\overset{\mathrm{Heat}}{\to }2\mathrm{BN}+3H_{2}O$$

### 2.4. Characterizations

The morphology and microstructure of MXene/BN composites were observed using field emission scanning FESEM, (FEI, Hillsboro, OR, USA, Verios G4) at 10 kV and 0.1 nA. Transmission electron microscopy (TEM, FEI USA, Talos F200S) was used to observe the fine structure of MXene/BN composites. X-ray diffraction (XRD, Shimadzu, Kyoto, Japan MAXima GLXRD-7000) with Cu Kα radiation and capillary attachment was employed for characterizing the crystal structure. The X-ray Photoelectron Spectroscopy (XPS) technique, utilizing Al Kα radiation (hν = 1486.6 eV, 15 kV, 10 mA) and offering an energy resolution of up to 0.45 eV (Ag 3d 5/2), was employed for analyzing the elemental composition and chemical configurations in the composites.

The electromagnetic parameters (real part ε′ and imaginary part ε″) of the dielectric constant were tested using a vector network analyzer (VNA, Rohde & Schwarz, Memmingen, Germany, ZNB40) in the frequency range of 0.5–18 GHz, following the typical coaxial method. The samples were prepared by mixing the as-prepared MXene/BN composite and paraffin in different weight proportions of 1:4. The prepared ring-like sample had an outer diameter of 7 mm and an inner diameter of 3 mm.

## 3. Results and Discussion

### 3.1. SEM Analysis

**Figure 1:**
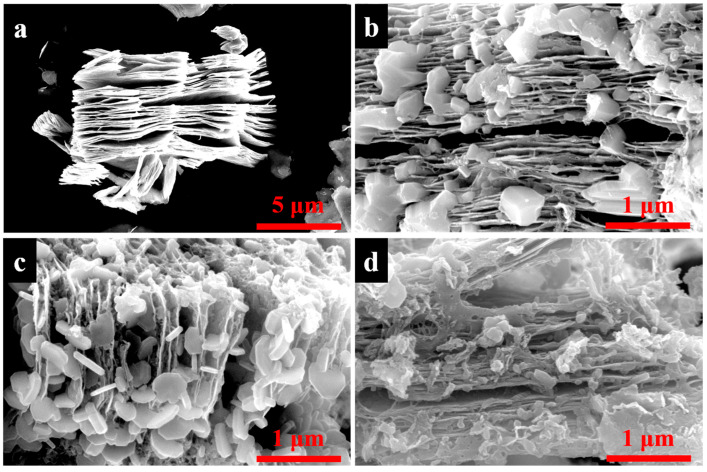
The SEM images of MXene and as-prepared MXene/BN-xyz composites: (**a**) MXene, (**b**) MXene/BN-251, (**c**) MXene/BN-201 and (**d**) MXene/BN-101 composites.

FTSEM characterization was used to examine the microstructure and morphology of MXene and MXene/BN composites. The SEM image in Figure 1a showed the presence of an accordion-like lamellar structure in MXene, providing numerous contact points for BN development. As depicted in Figure 1b, a small number of BN sheets grew on the MXene sheets at a ratio of 25:1, with the BN nano-sheets exhibiting irregular flakes and being ultra-thin. The microstructure of MXene/BN-201 composites is shown in Figure 1c. Compared with MXene/BN-251, the BN were noticeably increased and evenly distributed on the MXene sheets. Moreover, it can be observed that the BN nano-sheet protrusions had a nearly hexagonal shape and were attached to the surface of MXene, and the inner cavity of the MXene was filled with ultra-thin BN nanosheets. The morphology of MXene/BN-101 composites in Figure 1d showcases a stunning display. BN nano-sheets proliferated abundantly on the surface of MXene sheets, almost completely encapsulating them and giving rise to magnificent bulk protrusions.

### 3.2. TEM and EDS Analysis

**Figure 2:**
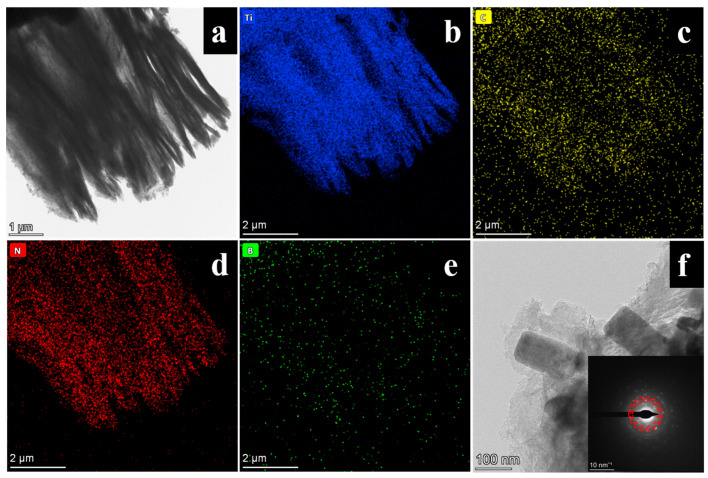
The TEM images of MXene/BN-201: (**a**) original image of MXene/BN-201, and (**b**–**e**) element mappings of the component. (**f**) Selected area electron diffraction pattern.

Figure 2a exhibits the original image of the composite MXene/BN-201. Ti and C elements can be clearly seen in Figure 2b,c. The distribution of Ti and C elements is consistent with the MXene main frame of the original diagram. From the distribution density, it conforms to the molar ratio of Ti_3_C_2_ (Ti:C = 3:2). It can be seen from Figure 2d,e that N and B were evenly distributed on the MXene sheets. The content of N was higher than that of B despite the ratio of 1:1 of BN. Because EDS is not sensitive to the B element test, the B element appeared to be less abundant. Figure 2f shows the transmission electron microscope image and selected area electron diffraction pattern of MXene/BN-201. It can be seen from the TEM image that the main body of Mxene is almost transparent under the electron beam, and the material has a certain thickness. In addition, three obvious crystal plane diffraction points can be seen from the SAED diagram, which are consistent with the three crystal faces of BN.

### 3.3. XRD and XPS Analysis

**Figure 3:**
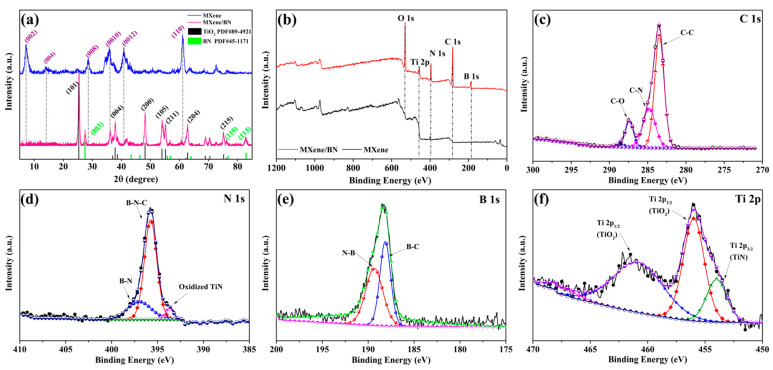
(**a**) XRD patterns of MXene and MXene/BN-201 composites; XPS survey spectra of the MXene/BN-201 composite was analyzed: (**b**) the full spectrum and (**c**–**f**) high-resolution spectra for B 1s, C 1s, N 1s and Ti 2p in the MXene/BN-201 composite.

The XRD thoroughly characterized the composition and crystal structure of MXene and MXene/BN-201. The blue line in the XRD pattern of Figure 3a showed the characteristic peaks of MXene. The diffraction peaks of the MXene were at 7.06°, 14.20°, 28.66°, 35.82°, 40.88° and 61.06°, corresponding to the crystal planes (002), (004), (008), (0010), (0012) and (110), which was consistent with the literature [45]. Additionally, the pink line in the XRD pattern of Figure 3a showed the characteristic peaks of MXene/BN-201. It could be seen clearly from the figure that there were three characteristic peaks: 2θ was at 26.72°, 75.95° and 82.12°, corresponding to the crystal surface (003), (110) and (113) of hexagonal boron nitride (PDF-#45-1171). However, there were two obvious broad characteristic peaks of TiO_2_ at 25.36°, 48.14° (PDF-#89-4921) and the characteristic peaks of MXene were shifted to different degrees in XRD patterns of MXene/BN-201. The possible reason was that parts of the MXene materials reacted to TiO_2_ by reacting with CO_2_ generated from the decomposition of urea at 900 °C.

The synthesized product was characterized by XPS to determine its element composition and configuration. Peaks in the full spectrum (Figure 3b) confirmed the presence of B, C, N, Ti and O elements through peaks of B 1s, C 1s, N 1s, Ti 2p and O 1s. Figure 3c–f show high-resolution spectra of C 1s, N 1s, B 1s and Ti 2p of the sample. As exhibited in Figure 3c, the C 1s peak at 284.86 eV could be deconvolved into three bands at around 284.71 eV, 285.01 eV and 287.45 eV, corresponding to three different chemical states of carbon atoms, which were bonded to C, N and O. The appearance of C-N peaks indicates that MXene was doped with N. As exhibited in Figure 3d, the N 1s spectrum of MXene/BN-201 was deconvolved into three peaks corresponding to N-B (397.02 eV), B-N-C (395.74 eV) and oxidized N-Ti (393.58 eV)[46], respectively. The presence of N-B indicates that BN was successfully recombined to MXene. Figure 3e shows the energy spectrum of B 1s, and the characteristic peaks of B-C and N-B appear at 188.13 eV and 189.34 eV. As shown in Figure 3f, the Ti 2p peak could be deconvolved into three bands at around 460.99 eV, 455.99 eV and 454.01 eV, corresponding to Ti 2p_1/2_ of TiO_2_, Ti 2p_3/2_ of TiO_2_ and Ti2p_3/2_ of TiN [47]. The findings provide additional evidence of the oxidation and nitrogen doping of MXene.

### 3.4. Discussion of Microwave Absorption Performance

The RL values of the composites prepared in this study were calculated and determined using the equations:

(4) $$Z_{in}=Z_{0}\sqrt{\frac{\mu_{r}}{\varepsilon_{r}}}\tanh\left[j\left(\frac{2\pi fd}{c}\right)\left(\sqrt{\mu_{r}\varepsilon_{r}}\right)\right]$$

(5) $$RL=20\mathrm{lg}\left|\frac{Z_{in}-Z_{0}}{Z_{in}+Z_{0}}\right|$$

where *Z_in_* represents the input impedance of the microwave absorbers and *Z*_0_ denotes the impedance of free space, approximately equal to 377 Ω. *ε_r_* refers to the relative complex permittivity (*ε_r_* = *ε*′ − *jε*″, where *ε*′ is the real part and *ε*″ is the imaginary part of complex permittivity), while *μ_r_* represents the relative complex permeability (*μ_r_* =*μ*′ − *jμ*″, with *μ*′ being the real and *μ*″ being the imaginary part of complex permeability). *f* stands for the frequency of incident electromagnetic waves, and *d* indicates the coating thickness of absorbers. *c* corresponds to a velocity constant equaling 3 × 10^8^ m/s.

**Figure 4:**
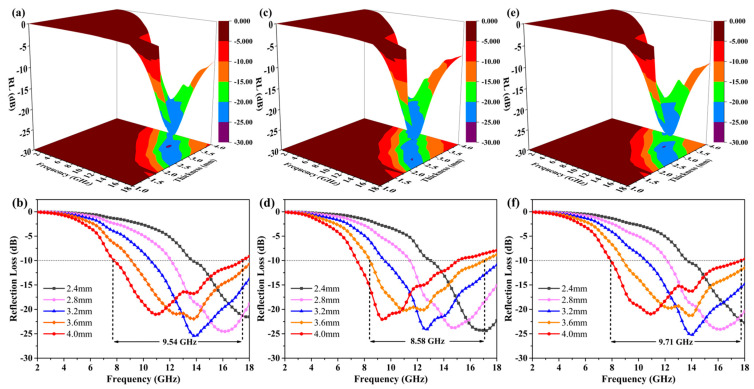
Optimum RL and 3D surface plots of MXene/BN-251 (**a**) and (**b**), MXene/BN-201 (**c**) and (**d**) and MXene/BN-101 (**e**,**f**).

The RL value diagram of the as-prepared absorber was calculated according to the Formulas (4) and (5) based on the electromagnetic parameter. The 3D color map and RL plots of MXene/BN-251, MXene/BN-201 and MXene/BN-101 are shown in Figure 4. For MXene/BN-251, the minimum RL value was −25.49 dB at the thickness of 3.2 mm with an effective absorbing bandwidth of 7.44 GHz (10.56–18.00 GHz). Although the minimum value of RL was −21.04 dB at the thickness of 4.0 mm, the effective absorbing bandwidth was as high as 9.54 GHz (7.85–17.39 GHz). It was obvious that the effective absorption bandwidth moved towards the low frequency range with the increase in thickness. The widest effective absorption bandwidth was obtained with the coating thickness of up to 4.0 mm. MXene/BN-201 (Figure 4c,d) delivered the minimum RL value of −24.39 dB at the thickness of 2.4 mm with an effective absorbing bandwidth of 4.99 GHz (13.01–18.00 GHz). However, the minimum value of RL was −20.13 dB at the thickness of 3.6 mm, and the effective absorbing bandwidth was up to 8.58 GHz (8.46–17.04 GHz). Figure 4e,f showed the MAP of MXene/BN-101, and the minimum value of RL was −20.94 dB at the thickness of 4.0 mm with an effective absorbing bandwidth of 9.71 GHz (7.94–17.65 GHz). To sum up, MXene/BN-101 had a wide effective absorbing bandwidth (9.71 GHz) from 7.94 GHz to 17.65 GHz based on the 3D surface diagram of the absorbing composite.

To better elucidate the disparity in MAP among the MXene/BN composite absorbents, it is imperative to comprehensively consider the electromagnetic parameters of the samples, which are closely associated with MAP. Since the as-prepared sample lacked magnetic properties, only dielectric parameters were considered in this study. The electromagnetic parameters were computed and deduced utilizing the provided equations:

(6) $$\varepsilon^{\prime }=\varepsilon_{\infty }+\frac{\varepsilon_{s}-\varepsilon_{\infty }}{1+\omega^{2}\tau^{2}}$$

(7) $$\varepsilon^{\prime \prime }=\frac{\varepsilon_{s}-\varepsilon_{\infty }}{1+\omega^{2}\tau^{2}}\omega \tau +\frac{\sigma }{\omega \varepsilon_{0}}=\varepsilon_{p}^{\prime \prime }+\varepsilon_{c}^{\prime \prime }$$

(8) $$tan\delta_{E}=\frac{\varepsilon^{\prime \prime }}{\varepsilon^{\prime }}$$

(9) $$\alpha =\frac{\sqrt{2}\pi f}{c}\times \sqrt{\left(\mu^{\prime \prime }\varepsilon^{\prime \prime }-\mu^{\prime }\varepsilon^{\prime }\right)+\sqrt{{\left(\mu^{\prime \prime }\varepsilon^{\prime \prime }-\mu^{\prime }\varepsilon^{\prime }\right)}^{2}+{\left(\mu^{\prime }\varepsilon^{\prime \prime }+\mu^{\prime \prime }\varepsilon^{\prime }\right)}^{2}}}$$

where *ε_s_* represents the material’s ability to store electric charge in a non-changing state. The dielectric constant at limit frequency state *ε*_∞_ characterizes the material’s behavior at high frequencies. *ε_0_* (dielectric constant at vacuum state) refers to the absence of any medium and serves as a reference point for comparison. *σ* denotes the conductivity of the material, which measures its ability to conduct electricity. The polarization loss component is represented by *ε_p_*″, while the conduction loss component is denoted by *ε_c_*″. Lastly, *α* represents the attenuation coefficient that quantifies signal degradation.

**Figure 5:**
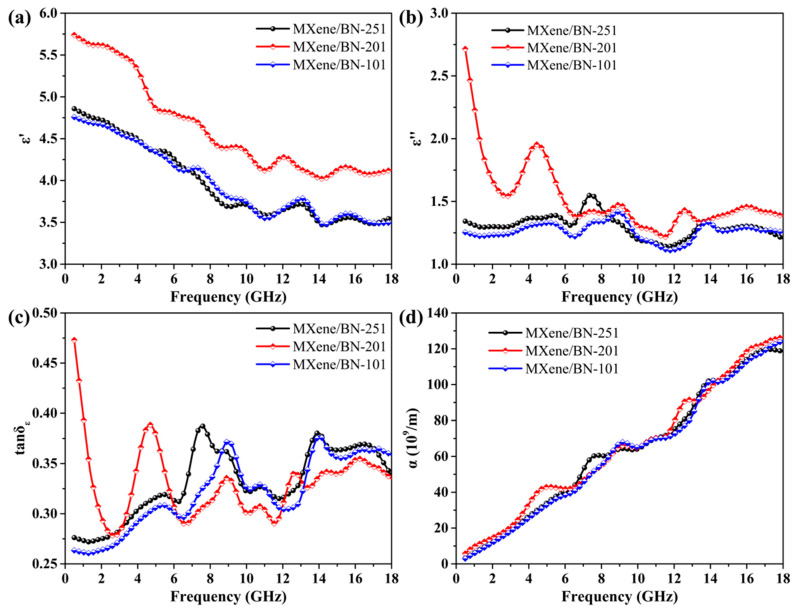
Electromagnetic parameters of Mxene/BN: (**a**) real part *ε*′, (**b**) image part *ε*″ of complex permittivity, (**c**) dielectric loss tangents tan*δ_E_*, (**d**) attenuation coefficient *α*.

The electromagnetic parameters of the samples were calculated as illustrated in Figure 5. *ε*′ represents the degree of polarization, which serves as a measure for the ability to store electromagnetic waves. As depicted in Figure 5a, the real part *ε*′ of the complex permittivity exhibited a decreasing trend with an increasing frequency of electromagnetic waves. This can be attributed to an accelerated periodic variation in the electric field, leading to material polarization and the generation of dipoles that are unable to keep up with these rapid changes [48]. Electric charges tend to accumulate at the contact interfaces, because of the disparate conductivity of MXene and BN, resulting in interfacial polarization when exposed to electromagnetic radiation [49,50,51]. The *ε*′ of MXene/BN-251, MXene/BN-201 and MXene/BN-101 varied from 3.47 to 4.86, 4.02 to 5.74 and 3.47 to 4.76 in the frequency range of 0.5–18 GHz. The *ε′* value of MXene/BN-201 was significantly higher than that of other materials. The FTSEM analysis revealed that BN with a regular hexagonal structure exhibited uniform growth both inside and outside the layers of MXene/BN-201, distinguishing it from the other two composite materials. This phenomenon led to an exacerbation of interfacial polarization originating from an increase in the contact interface. The *ε*″ represents the attenuation capability of electromagnetic waves resulting from the reorientation of electric dipole moments in response to an applied electric field. As exhibited in Figure 5b, the MXene/BN-201 composite exhibited the highest attenuation capability among all the MXene/BN composites, owing to the uniform incorporation of moderate BN in MXene/BN-201. Furthermore, MXene/BN-201 exhibited lower conductivity compared to other samples, which hindered the occurrence of the skin effect. The skin effect can cause incident microwaves to reflect on material surfaces, but in the case of MXene/BN-201, they were able to penetrate deeper into the material. Coating BN onto MXene/BN-101 obstructed its conductive path and resulted in reduced conductivity and weakened conductive loss. In Figure 5c, the tan*δ_E_* value was used to comprehensively evaluate the electromagnetic wave storage and attenuation capabilities of the materials. The variation in the value was a little complicated, and the MAP of MXene/BN-201 was superior to other Mxene/BN composites without considering impedance matching. The attenuation coefficient α exhibited an increase with the increasing frequency of electromagnetic waves in Figure 5d. By comparing the analysis of RL plots, it was observed that Mxene/BN-101 possessed a higher effective microwave absorbing bandwidth compared to other Mxene/BN composites, which aligns with our previous discussion on the attenuation coefficient. However, it would be unreasonable to solely consider attenuation.

**Figure 6:**
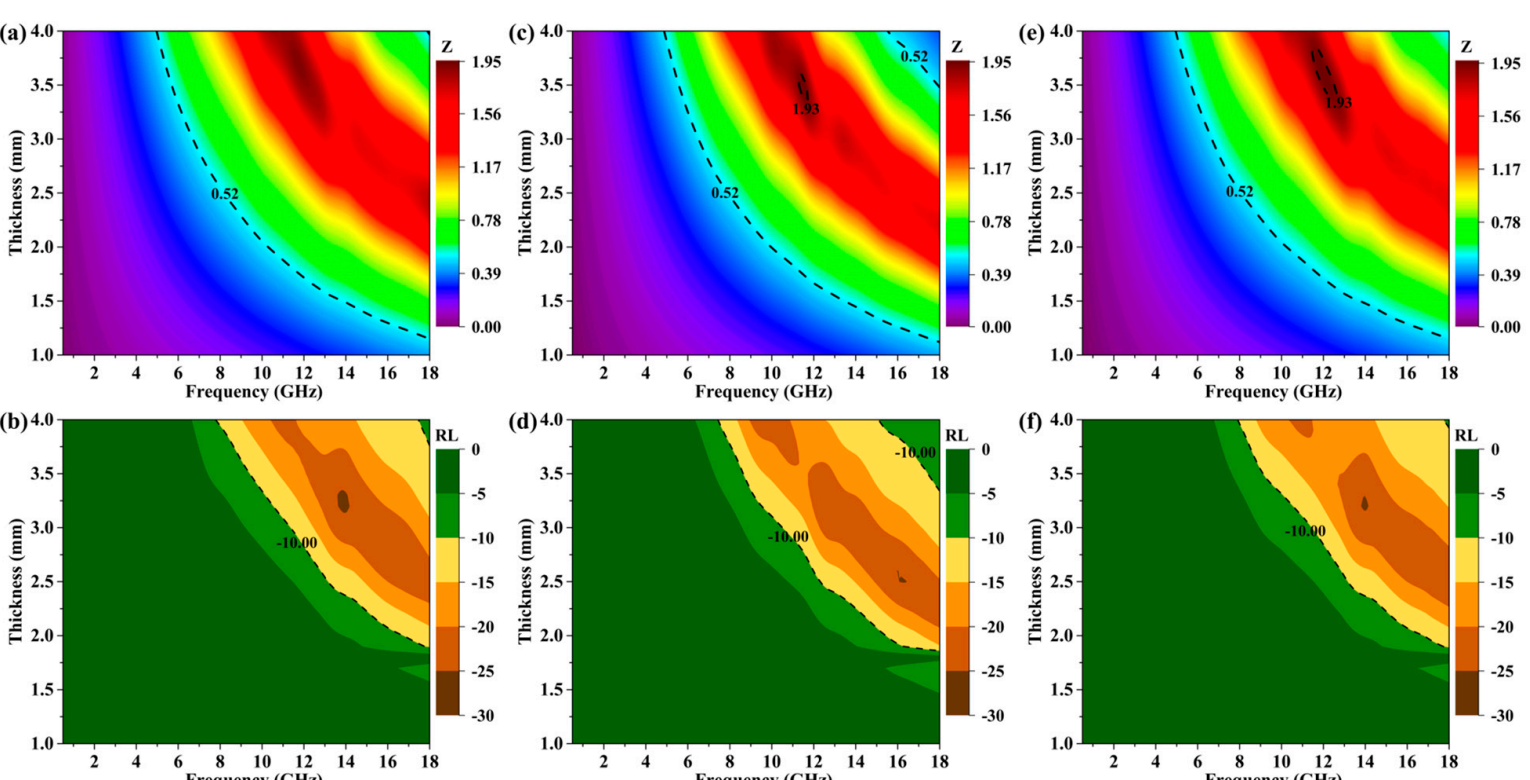
Z and RL contour maps for MXene/BN-251: (**a**) and (**b**); for MXene/BN-201: (**c**,**d**); and for MXene/BN-101: (**e**,**f**), respectively.

The factors that influenced the MAP included impedance matching and the attenuation coefficient. Additionally, an analysis of the impedance matching characteristics of the sample was conducted to further investigate the absorption mechanism within the MXene/BN composites. The presence of a well-matched impedance region can be observed within the range of 0.52 ≤ Z ≤ 1.93 in the Z contour map, when the MXene/BN composites exhibited sufficient attenuation properties for electromagnetic waves, corresponding to an effective absorption area (RL ≤ −10 dB) in the RL contour map [52]. Comparing Figure 6a and (b), it can be observed that the effective attenuation area was smaller than the region where 0.52 ≤ Z ≤ 1.93, but fell completely within the range of 0.7 ≤ Z ≤ 1.93 for MXene/BN-251, which could explain its lower α value compared to other composites. However, for MXene/BN-201 (Figure 6c,d), it is evident that the area of 0.52 ≤ Z ≤ 1.93 exceeded the corresponding effective attenuation area, like what was observed for MXene/BN-101 (Figure 6e,f). Furthermore, when comparing Figure 6a,c,e, it can be concluded that the order of the region with values between 0.52 and 1.93 was as follows: MXene/BN-201 < MXene/BN-101 < MXene/BN-251, indicating a significant correlation between impedance matching in the MXene/BN composites and the ratio of MXene to BN components used in their fabrication process. Therefore, based on the above discussion, it can be inferred that α plays a crucial role in determining the effective absorption capabilities in MXene/BN composites.

**Figure 7:**
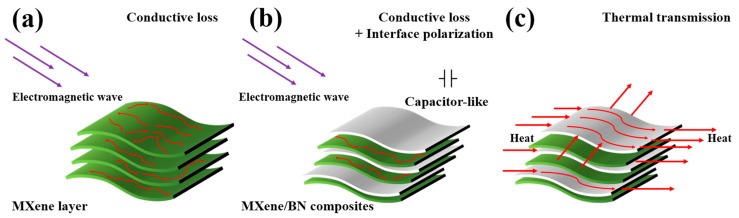
The EM wave-absorbing and heat-conduction mechanisms of MXene/BN-xyz composites (**a**–**c**).

The absorption mechanisms in as-prepared MXene/BN-xyz composites can be elucidated as follows: (i) the conductive loss in MXene serves as an effective mechanism for attenuating electromagnetic waves, as the interconnected network of conductors converts electromagnetic fields into electric currents and subsequently dissipates them as thermal energy [53,54] (as shown in Figure 7a); (ii) in Figure 7b, the numerous complex MXene-BN interfaces function as capacitors, capable of accumulating charges in response to electromagnetic fields, thereby inducing interface polarization; (iii) the presence of BN nanosheets hinders the formation of conductive networks in MXene, thereby reducing the properties of the absorber, specifically minimizing conductive losses while significantly increasing conductivity [55,56]; (iv) by incorporating BN nanosheets onto MXene, the conductive loss is mitigated, leading to a juxtaposition of reduced conductive loss and increased interface polarization loss, as depicted in Figure 7c.

## 4. Conclusions

A series of MXene/BN composites have been successfully synthesized and utilized for the first time in microwave absorption applications. The materials were characterized through XRD, XPS, SEM and TEM analysis, while the MAP was evaluated using network analysis techniques. Based on these investigations, the following deductions regarding electromagnetic wave absorption in these materials can be made:

(i) The compatibility between microwave absorption and the high temperature resistance of MXene/BN-xyz was assessed, showcasing the potential practical applications of MXene/BN-xyz in high temperature heating components due to its broad bandwidth (9.71; defined by RL < −10 dB from 7.94 to 17.65 GHz) and stability at 900 °C.
(ii) The competition between impedance matching and attenuation property arises upon the decoration of BN nanosheet onto MXene; the attenuation property plays a predominant role in the MAP.

## Figures and Tables

**Table 1:** The electromagnetic absorbing properties of MXene- or BN-based materials.

Materials	Thickness (mm)	Minimum RL(dB)	Bandwidth (RL< −10 dB) (GHz)	Refs.
MXene	1.40	−17.00	5.60	[38]
ZnO/Ti_3_C_2_T_x_	-	−26.30	4.00	[31]
SiC/Ti_3_C_2_T_x_	1.58	−41.70	3.36	[32]
MnO_2_/MXene	-	−18.80	4.56	[33]
CeO_2_/MXene	1.90	−47.27	4.22	[34]
TiO_2_/MXene	1.75	−58.30	4.08	[35]
CNT/MXene	2.65	−52.90	4.46	[39]
BN/Ni	1.70	-	3.00	[40]
TiN/BN	-	−16.74	3.26	[36]
BN/RGO	2.60	−48.90	4.20	[37]
FeSiAl/BN	2.14	−68.18	-	[41]
Ni/C	2.00	−45.00	4.50	[42]
SiC/C	1.50	−31.21	4.10	[43]
MXene/BN	4.00	−20.94	9.71	Our work

## Data Availability

Not applicable.
